# Action of Moral Nature on the Physical

**Published:** 1873-11

**Authors:** 


Action of the Moral Nature on the Physical.—Under this title, Dr. Cornette, (*Annales Medico-Psychologiques*, March, 1873,) gives two very interesting cases in which entire loss of hair, with change of voice, resulted from depressing emotions.

The first case was that of a perfectly healthy gardener, forty-five years of age, who, after undergoing considerable mental disquietude, through fear of impending poverty, for several weeks, found his hair suddenly growing white and at the same time falling out.

In a few days his head became almost completely bald, the few hairs which still remained in the form of a circle around the temples and occiput being straight, fine, sparsely scattered, and white as snow. His hair had previously been curly, abundant, and quite black. His beard rapidly became affected in a similar manner, and later the hair had entirely disappeared from all parts of the body.

At the same time, his voice, which before had been strong and full, became quite feeble and low in tone, and his whole appearance was that of a man of seventy-five. His health, however, was perfectly good.

Five years later his condition was nearly the same. He was quite melancholy, and but a few hairs, of a downy nature, remained on his body. His voice was still feeble.

The other case was that of a gentleman, fifty-six years of age, in political life and much harrassed by public and domestic anxieties.

One morning on awakening he found a large quantity of hair in his night-cap, and, running his hand through that which remained on his head, it came out by handfuls.

Within a very short time he had lost not only all the hair on his scalp, but also that on the chin, and, finally, on all parts of the body.

After six years this gentleman's condition remained the same. His skin had returned to the smoothness of infancy; one would never believe it had been otherwise.

His general health remained good, but, like the previous case, his voice was nearly lost at first, and even after several years was still quite feeble.

